# Retrospective review of patients with atrial fibrillation: does pulmonary vein isolation make a difference?

**DOI:** 10.1186/1532-429X-14-S1-T3

**Published:** 2012-02-01

**Authors:** Ronald B Williams, Sahadev T Reddy, Mark Doyle, June Yamrozik, Vikas K Rathi, Robert W Biederman

**Affiliations:** 1 Allegheny General Hospital, Pittsburgh, USA

## Summary

The success rate in patients with atrial fibrillation that were treated with pulmonary vein ablation is suboptimal. In retrospective review, those PVI patients that sustained normal sinus rhythm showed improvement in LV function and reverse remodeling of mitral apparatus when compared to those that had no response.

## Background

It has been documented in the literature that patients with chronic or recurrent atrial fibrillation (AF) undergo left atrial remodeling as well as mitral annulus changes presumably related to volume overload within the atrium. We wish to show that cardiac MRI (CMR) imaging can be utilized for pre and post PVI for AF evaluation of atrial and LV annular remodeling.

## Methods

Ninety four (94) patients, with AF, (19 females; 59±9.9 years; 57 males, 49±9.7 years with sustained normal sinus rhythm (NSR) and eighteen (18; 4 females ±6 years; 14 males, 56± 9 years) post-PVI recurrent or failures), underwent CMR using standard, SSFP imaging, (GE Excite HD, 1.5, Milwaukee, WI). Routine VLA, HLA, LVOT views were obtained as well as MRA for 3D viewing of pulm.

## Results

All 94 patients were reviewed, 76 (81%) of those with successful PVI resulting in sustained NSR, showed significant improvement in their mitral apparati and MR severity. Those AF patients that didn’t respond to PVI, showed no significant changes in either MR reduction or geometric remodeling (Table 1 and 2).

**Table 1:** NSR Patients Pre/Post

n=76			
CMR Cardiac Parameter	Pre PVI	Post PVI	P Value

LA Volume (ml)	230±70	199±66	0.01
LVEF%	57±10	60±6	0.01
MR Severity (Mean)	0.78±0.8	0.51±0.9	0.01
Mitral Annulus Diameter (mm)	34.5±3.9	32.6±3.9	<0.001
Mitral Tenting Area (mm2)	169.7±55.9	138.9±40.6	<0.001
Mitral Tenting Height (mm)	8.0±2.0	7.2±1.8	<0.001
Mitral Tenting Angle	131.2±11.6	130.8±9.7	NS

**Table 2:** Patients that did not Respond to PVI

n=18			
CMR Cardiac Parameter	Pre PVI	Post PVI	P Value

LA Volume (ml)	207±55	183±85	0.05
LVEF%	59.3±10	60.9±8.6	NS
MR Severity (mean)	1.1±0.8	0.7±0.9	NS
Mitral Annulus Diameter (mm2)	33.1±4	32.9±3.7	NS
Mitral Tenting Area (mm2)	154±41.8	143.8±45.4	NS
Mitral Tenting Height (mm	6.9±1.9	7.0±1.8	NS
Mitral Tenting Angle (degrees)	131.2±11.6	130.8±9.7	NS

## Conclusions

Patients treated with PVI for AF demonstrate significant improvement in LV function and secondary improvement in LV/atrial reverse remodeling with subsequent improvement in MR only in responders (NSR). Patients who failed PVI show no significant improvement in degree of MR or any LV/atrial metrics.

## Funding

Internal.

